# Construction of a nasopharyngeal carcinoma 2D/MS repository with Open Source XML Database – Xindice

**DOI:** 10.1186/1471-2105-7-13

**Published:** 2006-01-11

**Authors:** Feng Li, Maoyu Li, Zhiqiang Xiao, Pengfei Zhang, Jianling Li, Zhuchu Chen

**Affiliations:** 1 Key Laboratory of Cancer Proteomics of Chinese Ministry of Health, Xiangya Hospital, Central South University, Changsha, China; 2 Cancer Research Institute, Central South University, Changsha, China

## Abstract

**Background:**

Many proteomics initiatives require integration of all information with uniformcriteria from collection of samples and data display to publication of experimental results. The integration and exchanging of these data of different formats and structure imposes a great challenge to us. The XML technology presents a promise in handling this task due to its simplicity and flexibility. Nasopharyngeal carcinoma (NPC) is one of the most common cancers in southern China and Southeast Asia, which has marked geographic and racial differences in incidence. Although there are some cancer proteome databases now, there is still no NPC proteome database.

**Results:**

The raw NPC proteome experiment data were captured into one XML document with Human Proteome Markup Language (HUP-ML) editor and imported into native XML database Xindice. The 2D/MS repository of NPC proteome was constructed with Apache, PHP and Xindice to provide access to the database via Internet. On our website, two methods, keyword query and click query, were provided at the same time to access the entries of the NPC proteome database.

**Conclusion:**

Our 2D/MS repository can be used to share the raw NPC proteomics data that are generated from gel-based proteomics experiments. The database, as well as the PHP source codes for constructing users' own proteome repository, can be accessed at .

## Background

The completion of human and other model-organism genome projects has provided a sequence infrastructure to allow an improved understanding of the dynamic processes of cellular signaling, regulation, and metabolism. Although all cells contain the complete genome, only a fraction of the genes are expressed within a given cell. Under different conditions or within different tissues of the same organism, a specific set of proteins are expressed and/or post-translationally modified to carry out the special function of the cell [1-3]. The term 'proteome' is a hybrid of "PROTEin" and "genOME" and it refers to the entire protein components, along with all covalent protein modifications in a selected cell. With the arrival of the post-genomic era, functional genomics has become a new focus of biological research, and proteomics has emerged as a promising field for assessing global protein function [4].

In order to understand the roles of different proteins played and to dissect protein-protein interaction networks, high-throughout methodologies are being applied in the emerging field of proteomics. As a result, large amounts of experimental data have been generated by high-throughout proteomics methodologies, such as large-scale two-hybrid systems, high-throughout mass spectrometry technology, and multi-dimensional chromatograph. Meanwhile, with the volume of proteomics information increasing rapidly, there has been a great need for a public proteomics repository and for exchanging these raw proteomics experiment data between labs [5]. The raw experiment data is usually generated by different instruments, laboratories and methods, thus it is still difficult to exchange the raw proteomics data directly.

Recently, a new special organization called PSI (Proteomics Standards Initiative) was founded at the HUPO (Human Proteomics Organization) meeting in Washington D.C. USA to define community standards for data representation in proteomics to facilitate data comparison, exchange and verification [6]. Since the raw proteomics experiment data produced in our lab and the technologies used in most proteomics labs are still based on the 2D/MS systems, we intended to focus on the exchange of the raw proteomics data produced by 2D/MS systems with general proteomics format.

Currently, there have been some XML models developed as standards with relevance to the whole of proteomics such as PEDRo, HUP-ML and AGML [7-11]. Among these models, PEDRo and HUP-ML are the two popular XML models used to process raw proteomics data. PEDRo was developed by a group led by Prof. Norman Paton, which takes into account many aspects of gel-proteomics data compared with other XML models, such as mzXML, mzData and mzIdent, which emphasize more specifically for mass spectrometry data [12,13]. HUP-ML is another proteome- analysis-oriented format based on XML, and it was proposed by Kamijo et al. at the 2002 AOHUPO XML Workshop. The data model of HUP-ML is based on the classical 2D/MS systems and it can be used by most labs. Here we adopted the HUP-ML editor as the data capture software and HUP-ML data model for our NPC proteomics repository.

NPC is one of the most common cancers in southern China and Southeast Asia, which demonstrates remarkable geographic and racial differences in incidence. Public proteome repository is the infrastructure to study the complicated mechanisms of caner. Although there are many cancer proteome databases, there has been no NPC proteome database to our knowledge. In this paper, we used HUP-ML editor to collect the raw NPC proteomics data inclued the experiment results and experiment conditions. Then, these XML documents were imported into Xindice database, and PHP was used to pass the query request from web clients to database manage system (DBMS) and to convert the query results back to the clients with HTML format. The PHP source codes can be downloaded from our website  to construct a user's own proteome repository.

## Results

**Figure 1:**
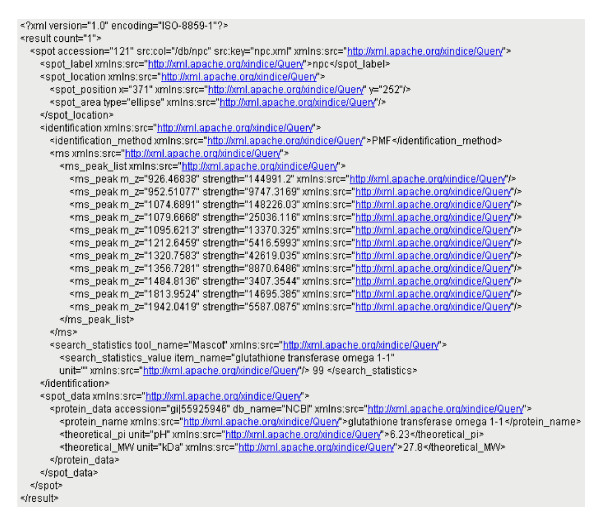
**Example of Spot Section processed by Internet Explore**. One NPC 2D/MS repository query result displayed in Internet Explore without transforming with Sablotron XSLT processor. The root of the query result XML document was result tag. Every query record would be inserted among spot tag.

**Figure 2:**
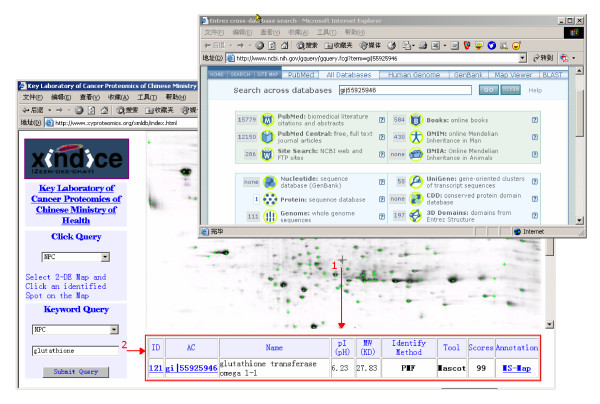
**An example of clicking a clickable spot or keryword query**. An example of query result was displayed in the client browser. Two methods to query the NPC 2D/MS repository were provided, one is to click the clickable spot and the other is to query with keyword. The user could see the position of spot on the gel through the hyperlink of ID and other information about the spot such as pI, MW, accession number, scores of Mascot or other database search software and etc. Through the hyperlink of accession number in the NCBI, the user could get more information about this protein directly without searching again in NCBI.

An example of an Xpath query result of the NPC gel-proteomics experiment data in the NPC 2D/MS repository is shown in Figure 1, the architecture of this repository is shown in Figure 5. To retrieve the exact information of an identified spot, two choices were provided to query the information. One method is through the text input to query the database with a NCBI accession number, a protein name or synonym name, or gene name. Another query method is through clicking the clickable spots on the 2-DE gel maps. Both query methods were based on the Xpath query. The results of an Xpath query are returned as a XML document. To display the query results in a readable format, a transform must be done with an XSLT processor before output to the client browser. An example of Sablotron XSLT processor transformed result is shown in Figure 2. In the upper right frame, the returned spot result has been shown with red cross on the 2-D gel image, and at the same time the detail protein information of the queried spot is shown in the bottom right frame. Another query method is to directly click a spot on the 2-D gel image. If the spot has been identified in the experiment, the detail protein information will be displayed in the bottom right frame. Both query methods allow users to access the related functional annotation information of the protein in the NCBI database through hyperlink.

**Figure 3:**
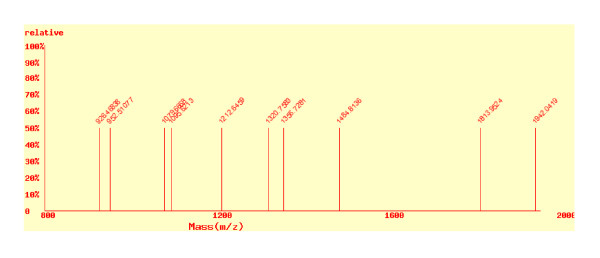
**PMF map of monoisotopic peak list**. Through the PHP scripts in our web sever, the mimic PMF map could be displayed in the client browser. As shown in the PMF map of glutathione transferase omega 1-1, all monoisotopic peaks could be displayed and used to compare with the user's PMF map.

In our NPC proteomics repository, peak list of the monoisotopic peaks of every peptide mass fingerprint (PMF) maps is extracted with Mascot Distiller and saved as mgf file. All mgf files have been changed to text files and imported into HUP-ML documents. When the users click the hyperlink of MS-Map of the identified spot, the DBMS queries that node and extracts the monoisotopic peaks from ms_peak_list tag to PHP, which will then be transformed to a mimic PMF map. By this method, the mimic of raw PMF maps can be shared by everyone without limitation of file format defined by the mass spectrometry manufacturer. Figure 3 shows the PMF map of an identified protein glutathione transferase omega 1-1 generated by monoisotopic peak list.

## Discussion

There are currently two types of DBMS used to store proteomics experiment results, there are relational database manage system (RDBMS) and XML database system. Currently, most public 2D/MS databases adopt SWISS-2DPAGE database or free RDBMS, such as MySQL, to store and manage their data. The SWISS-2DPAGE database is based on the Make2ddb software of SIB (Swiss Institute of Bioinformatics). The backend database system of Make2ddb is PostgreSQL RDBMS. Although the SWISS-2DPAGE database is well established, certain important experimental information and raw data still can not be integrated into database, such as the condition of protein separation and identification, the detailed descriptions of experimental samples, the raw mass spectrometry maps and etc. If researchers use other free RDBMS, they have to spend a substantial effort on designing and optimizing the database for the information. The advantage of RDBMS is that it can be used easily to store, manage, and query the structural information because of its specially designed structural and relational model. Nevertheless, complicated data structure in the proteomics data integrated with HUP-ML model makes it difficulty to construct a proteomics repository with RDBMS because of some problems in mapping hierarchical structure to relational schema. In addition, if we use RDBMS as back-end and map the proteomics data into tables, such DBMSs force us to fragment our data into many pieces to satisfy the third normal form requirement. The fragmentation can also impose efficiency problems, as a query can cause the DBMS to perform many joins to reassemble the fragments into the original data.

XML technology is the next generation of the Internet language. It has powerful capability in exchanging data, and XML technology is particularly well suited to represent biological data and methods and is presently the consensus choice in most areas including proteomics because XML is highly flexible and XML provides an open framework for defining standard specifications [11,14-16]. As web services grow rapidly, XML flourish more andmore in data exchanging and sharing, and has resulted in two XML-based new database technology: Native XML DBMS (NXD) and XML-Enabled DBMS (XED). With NXD, there is no need to map the special proteomics schema to RDBMS. Xindice is an Open Source Native XML database developed by Apache, which is a software foundation that promotes the construction of web-based tools and standards. Compared with other Open Source XML databases, such as eXist and xmldb, we think Xindice may be more stable with better compatibility and technical supports. Therefore, we decided to adopt the NXD database Xindice to store, manage, and query the collection of raw NPC proteomics experiment data.

PEDRoDB is another new database system for storing, searching, and disseminating experimental proteomics data, and it stores the raw proteomics data as XML format with Xindice. PEDRoDB is a database system based on raw data capture software Pedro, which has been developed to encode laboratory data and to generate an XML-based PEML (Proteomics Experiment Mark-up Language) file based on PEDRo model for local storage or submission to a database. Unlike 2D/MS databases based on Make2ddb, which emphasizes more on gel annotations, the PEDRoDB database was designed to provide more information, allowing detailed comparisons of the ways by which the results were obtained [10]. However, PEDRoDB is not available for downloading at least as of our writing.

The HUP-ML document uses a flat file structure and it can be treated as a database or a table of RDBMS in some sense. The related XML document can be directly put into the same directory and processed by the file manage system. But the functionality of this method is still insufficient, as it cannot provide the merit of a database, such as event security rescue mechanism, parallel control, and high efficient indexing and querying. Therefore, by deploying the NXD to process the HUP-ML documents, the whole system can be more efficient and secure.

Xindice is an open source native XML database, featuring efficient querying based on Xpath, XUpdate support, and tight integration with existing XML development tools. However, Xindice is subjected to the common limitations of NXD because of its short existence compared with RDBMS, and not too many NXD-supported technologies and applications have been available.

Both PEDRo and HUP-ML represent the current efforts in using XML technology to exchange the raw proteomics data. At present, it is a good choice to use an existing effort, PEDRo or HUP-ML, as a starting point for system design rather than a new one. To choose a raw proteome data capture software, we think that the annotation of gels may be more useful than detail description of experiment conditions. Therefore, we select the HUP-ML to intergate the different source information of gel-proteomics data.

Peptide mass fingerprint map and tandem mass spectrometry are currently the two most commonly used technologies in proteomics for protein identification. Because the mass spectrometry used in different labs were made by different manufacturers, the raw PMF maps and MS/MS maps generated by different equipment use different file formats which can only be opened with the special software from the mass spectrometry manufacturers. This has greatly increased the difficulty for exchanging the raw mass spectrometry data. A standard peak list format, such as mzData, provided by PSI requires many agreements from the original software provided by the spectrometry manufactures to third party software developing companies and will be implemented into next version [17]. We extracted the monoisotopic peak lists, which comprise the m/z data extracted from the raw maps, and imported it into NPC repository. Through the monoisotopic peak lists, user could view the raw PMF maps and compare with the user's own MS maps.

Although Xindice is fit for serving as backend of NPC repository, some factors should be taken into account in order to improve the performance of database query. Database indexes are a powerful technology to improve the efficiency of database querying. Suppose the browsers commonly use the protein name and NCBI accession to query database, here we adopt element "protein_name" and "protein_data" element's "accession" attribute to index the NPC collection but it costs almost the same time with no database index. Unexpected observation might be due to a bug with Xindice or a problem with our implementation. The size of the data file is another affecting factor. Now the size of a file integrating all the data of 216 spots into Xindice is about 600 KB, which can be considered as a medium sized file compared to the 5 Mb Xindice file limit. Because Xindice was specifically designed for managing many small to medium sized documents, it is not a good way to integrate everything into one file even though the present sizes of NPC documents are still acceptable. Integrating everything into one big file increases the file complexity and the required more time for database query, especially as the identified spots increase. We think one solution is to extract the data of each spot into a single file and import all these files into one collection when the data expand, and this is also an important optimization step involved in constructing our NPC repository. Although the benchmark test of database has not been performed, it is better to be done before optimizing and tuning the database.

## Conclusion

With our PHP source code, 2D/MS experimental data can be delivered over the World Wide Web in an easily understandable format. One advantage of our platform inherent to representing information as alphanumeric strings is that the data can be easily stored and transmitted between different computer platforms and applications using the emerging XML technology, which is particular suitable for the development of proteomics web-services. Another advantage of PHP plus XML is that this platform can be rapidly constructed and it can greatly decrease the efforts on databases designing, storing and exchanging between different labs by using the same standard formats. Our website provide information more focused on the results of 2D/MS experiments, such as identified spots, 2-DE maps, Peak Lists.

## Methods

### Test materials and XML source files

Fresh nasopharygeal biopsy specimens obtained from a pool of 5 patients presented with symptoms possibly indicative of NPC at the Xiangya hospital, Hunan province, China, were used in this study. The specimen was immediately frozen in liquid nitrogen after excision and flushing out of blood, and stored at -80°C until analysis while were histological proven low-differentiated squamous cell carcinoma. The protocols for sample preparation, 2-DE and spot identification by mass spectrometry were the same as previously described [8]. Mascot Distiller was used to get the monoisotopic peaks from the raw mass spectrometry files. Then the monoisotopic peaks were used to search the MSDB database with Mascot search engine [18]. The searching parameters were set up as follows: Homo sapiens as taxonomy selection; the mass tolerance was ± 100 ppm, numbers of missed cleavage sites were allowed up to 1, the fixed modifications were selected as carbamidomethyl (cysteine), the variable modification was selected as oxidation (methylation) or none. All experiment conditions and experiment results, such as 2-DE gel images, peak lists of peptide mass fingerprint, and protein information of the identification spots, were integrated into XML documents with HUP-ML editor and validated by HUP-ML editor with HUP-ML schema hup-ml.dtd. The schema of XML document can be downloaded from JHUPO [19]. In the 2-DE gel map of NPC, 216 spots were identified by MALDI-TOF mass spectrometry and 41 spots among these spots were identified by MALDI-TOF and Q-TOF mass spectrometry either. (manuscript submitted)

### Software environment

**Figure 4:**
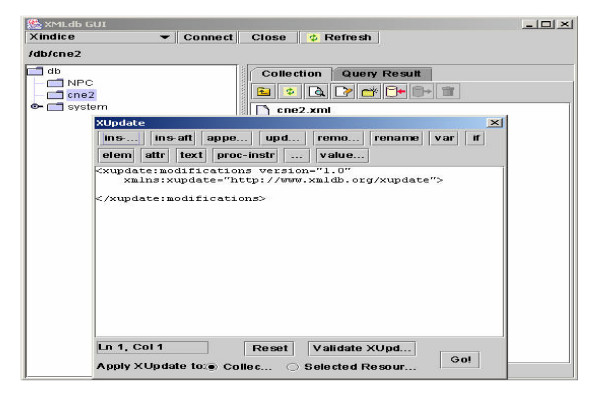
**The interface of XUpdate**. With the interface of XUpdate in the XMLDBGUI, the data of NPC proteomics repository could be updated easily.

We use Compaq compliant 370 running Windows 2000 Professional as our computer server. We use J2SDK1.4.2 java development environment, Apache1.3.29 as web server, and PHP sever to take the requests of client's browser and return the search results of XML documents to the browser. Xindice-1.0 as a native XML database was installed on the database sever to store and manage the collection of raw proteomics XML documents, to process the query request, and to update the experiment results using XUpdate. Xindice-XMLRPC 0.6 was installed on the web server to serve as a simple XML-RPC access API (Application Program Interface) to manipulate the Xindice database. XMLDBGUI was downloaded from DSTC and installed on the web server to monitor the Xindice status on the local computer and update the repository with XUpdate function at the local machine as shown in Figure 4[20].

### Architecture for exchanging proteomics data

**Figure 5:**
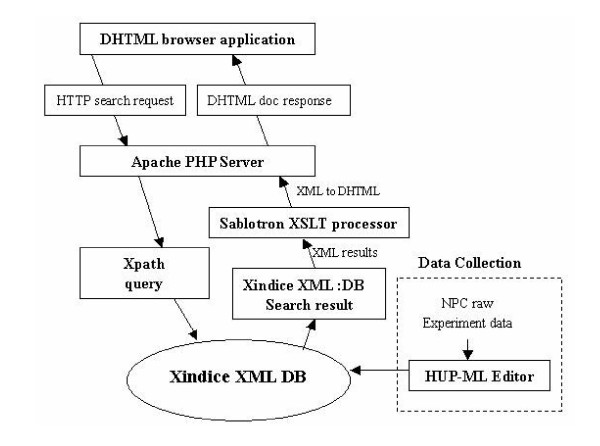
**Architecture of exchange proteomics data**. The figure showed the architecture of NPC repository and information flow of querying NPC 2D/MS repository.

The XML repository has been designed according to the rules proposed by Appel that were successfully used in constructing the 2D/MS database of ExPASy [4]. Unlike the Make2ddb package, which is based on the postgreSQL RMDB, the XML repository is based on native XML database. Different source information including IEF condition, SDS-PAGE condition, 2-DE gels image and spots identification information including protein name, peak lists of peptide mass fingerprint and MS/MS tags was first collected into one XML document with HUP-ML editor. Then, different HUP-ML documents were imported into the Xindice XML database without modifying the schema. The architecture for exchanging proteomics data is shown in Figure 5. To manipulate with the Xindice database, XML-RPC (remote procedure calling) was used as the API of web service.

## Authors' contributions

FL and MYL implemented the software and coordinated the data capture activity. PFZ and JLL conducted or led experimental activities that generated the data in the database, and contributed to feedback on querying the database. ZQX and ZCC oversaw the database design and development activity, and the latter led the write-up.
